# Flavonoid Compounds Contained in *Epimedii Herba* Inhibit Tumor Progression by Suppressing STAT3 Activation in the Tumor Microenvironment

**DOI:** 10.3389/fphar.2020.00262

**Published:** 2020-03-18

**Authors:** Cheng Pan, Yukio Fujiwara, Hasita Horlad, Daisuke Shiraishi, Toyohisa Iriki, Jyunko Tsuboki, Tsuyoshi Ikeda, Yoshihiro Komohara

**Affiliations:** ^1^Department of Cell Pathology, Graduate School of Medical Sciences, Faculty of Life Sciences, Kumamoto University, Kumamoto, Japan; ^2^Department of Orthopedic Surgery, Graduate School of Medical Sciences, Faculty of Life Sciences, Kumamoto University, Kumamoto, Japan; ^3^Department of Respiratory Medicine, Graduate School of Medical Sciences, Faculty of Life Sciences, Kumamoto University, Kumamoto, Japan; ^4^Department of Obstetrics and Gynecology, Graduate School of Medical Sciences, Faculty of Life Sciences, Kumamoto University, Kumamoto, Japan; ^5^Faculty of Pharmaceutical Sciences, Sojo University, Kumamoto, Japan

**Keywords:** *Epimedii Herba*, STAT3, flavonoid, macrophage, tumor cells

## Abstract

M2-like tumor-associated macrophages (TAMs) in the tumor tissues promote tumor progression by various mechanisms and represent possible targets of antitumor therapy. In the present study, we tested whether compounds from *Epimedii Herba* inhibit macrophage polarization to the M2/protumorigenic phenotype and prevent tumor progression, using human monocyte-derived macrophages (HMDMs) and an animal sarcoma model. Four *Epimedii Herba*-derived flavonoid compounds, namely, limonianin, epimedokoreanin B, icaritin, and desmethylicaritin, inhibited CD163 expression and interleukin (IL)-10 production, which are known M2 markers, suggesting that these compounds inhibit M2 polarization. Among these compounds, epimedokoreanin B and limonianin suppressed STAT3 activation in HMDMs. Notably, epimedokoreanin B also suppressed cell proliferation by blocking STAT3 activation in Saos-2 human sarcoma and LM8 mouse sarcoma cell lines. Furthermore, oral administration of epimedokoreanin B inhibited tumor growth in an LM8 tumor-bearing murine model. These results indicate that *Epimedii Herba* and *Epimedii Herba-*derived compounds, such as epimedokoreanin B, may be potentially new agents that can be used for the treatment and prevention of various malignant tumors. They may also be promising compounds for targeting the tumor microenvironment by inhibiting M2 polarization of the TAMs.

## Introduction

The heterogeneity of macrophage activation plays an important role in innate immunity and is involved in the pathogenesis of various diseases. Initially, in the 1990s, several differences were found between interferon (IFN)-γ-stimulated macrophages and interleukin (IL)-4-stimulated macrophages, and these were called classically and alternatively activated macrophages, respectively (Stein et al., 1992). Later, based on arginine metabolism, macrophages were characterized to be of two phenotypes, namely, Th1- and Th2-like phenotypes, also described as M1 and M2 phenotypes, respectively (Mills et al., 2000). Now these concepts have been blended, and the M1/M2 paradigm is well known. M1-like macrophages are proinflammatory, characterized by high levels of IL-1β, tumor necrosis factor-α (TNF-α), and IL-23 production, and are involved in the elimination of invading microorganisms. In contrast, M2-like macrophages are anti-inflammatory and are mainly involved in immunosuppression and tissue remodeling. M2-like macrophages produce high levels of IL-10 and low levels of IL-12 and express special receptors such as the mannose receptor (CD206), class A scavenger receptor (SR-A, CD204), hemoglobin scavenger receptor (CD163), and DC-SIGN (CD209) (Mantovani et al., 2002, 2004, 2013; Martinez et al., 2006; Komohara et al., 2014, 2015). The phenotype of macrophages is highly plastic and can change between the M1 and M2 phenotypes depending on the types of stimulii (Porcheray et al., 2005).

The presence of tumor-associated macrophages (TAMs) in tumor tissues is closely associated with the development of the tumor microenvironment (Lewis and Pollard, 2006; Komohara et al., 2014). Clinically, a higher number of infiltrating M2-like TAMs is generally associated with poor prognosis and higher grade of malignancy in patients with various cancers, including glioma, sarcoma, and lymphoma (Espinosa et al., 2009; Clear et al., 2010; Komohara et al., 2011b, 2014; Kurahara et al., 2011; Takeya and Komohara, 2016). In addition, *in vitro* studies have shown that M2-like TAMs can promote tumor cell proliferation, angiogenesis, invasion, and metastasis by secreting several kinds of protumor cytokines and chemokines (Mantovani et al., 2002; Pollard, 2004; Sica and Bronte, 2007). Therefore, inhibiting polarization of macrophages to the M2 phenotype is considered a promising approach for antitumor therapy.

In the present study, we tested whether compounds derived from *Epimedii Herba* inhibit the polarization of macrophages to the M2 phenotype using a screening system to identify compounds that inhibit tumor progression.

## Materials and Methods

### Cells and Cell Culture

Saos-2 (a human sarcoma cell line) and LM8 (a mouse sarcoma cell line) cells were purchased from the RIKEN Cell Bank (Tsukuba, Japan) and cultured in RPMI 1640 supplemented with 10% fetal bovine serum (FBS), and 100 μg/mL penicillin and streptomycin. We had previously established a high metastatic-potential cell line, LM8 clone 5 (Horlad et al., 2013), and we used this clone for the *in vitro* and *in vivo* studies. These cells were regularly tested and found to be negative for *mycoplasma* contamination.

Peripheral blood mononuclear cells were obtained from healthy volunteers, and written informed consent was obtained from all the donors. All protocols using human materials were approved by the Kumamoto University Review Board (No. 486) and were conducted in accordance with the approved guidelines. Monocytes were isolated using Lymphoprep^TM^ and then stimulated with GM-CSF (5 ng/mL) or M-CSF (100 ng/mL) for 7 days to differentiate them into human monocyte-derived macrophages (HMDMs). HMDMs were cultured in DMEM supplemented with 2% FBS, and 100 μg/mL penicillin and streptomycin.

### General Procedure

The NMR spectra were measured with a JEOL ECA 500 NMR spectrometer. Preparative HPLC was performed using a SIMADZU LC-20AT pump, JASCO 830-RI detector, Sugai U-620 column heater, and column of COSMOSIL 5C_18_ AR-II (5 μm, ϕ10.0 × 250 mm, Nacalai Tasque Inc., Kyoto, Japan), SunFire Prep C_18_, X-Bridge Prep C_18_ (5 μm, ϕ10.0 × 250 mm, Waters Co., MA, United States) with a flow rate of 2.0 mL/min and column temperature of 40°C. TLC was performed on pre-coated silica gel 60 F_254_ (Merck Ltd., Frankfurter, Germany) and detection was achieved by spraying with 10% H_2_SO_4_ followed by heating. Column chromatography was carried out on MCI gel CHP-20P (Mitsubishi Chemical Co., Tokyo, Japan), Sephadex LH-20 (GE Healthcare Bioscience Co., Uppsala, Sweden), μ-Bonda Pak C_18_ (Waters Co., MA, United States), and silica gel 60 (230-400 mesh, Merck Ltd., Frankfurter, Germany).

### Plant Materials

*Epimedii Herba* (lot number: C1S1504) was purchased from Uchida Wakan-yaku Co. Ltd. (Tokyo, Japan) according to the specifications in the Japanese Pharmacopeia, which permitted the use of *Epimedium* spp. including *Epimedium pubescens* Maximowicz, *Epimedium brevicornu* Maximowicz, *Epimedium wushanense* TS Ying, *Epimedium sagittatum* Maximowice, *Epimedium koreanum* Nakai, *Epimedium grandiumum* Morren var. *thunbergianum* Nakai, and *Epimedium sempervirens* Nakai. A voucher specimen was deposited at the herbarium of the Faculty of Pharmaceutical Sciences, Sojo University, Japan (SJU1103).

### Extraction and Isolation

The aerial parts of *Epimedium* spp. (3.0 kg) were extracted twice with MeOH by sonication for 6 h (30 min × 12) at room temperature (20–25°C). The extract was concentrated under reduced pressure to obtain a residue (485.0 g). The residue was partitioned between *n*-Hexane and 80% MeOH, and the 80% MeOH layer was concentrated to give a residue (408.1 g), which was loaded onto a MCI gel CHP-20P column [ϕ50 × 300 mm; eluted with H_2_O-MeOH gradient (0, 50, 100%; each MeOH%; 1.5 L of each gradient solution)] to give three fractions. The second fraction (46.5 g, eluted with 50% MeOH) was further applied to a MCI gel CHP20P column [ϕ50 × 300 mm; eluted with H_2_O-MeOH gradient (50, 60, 70, 80, 100%; each MeOH%; 1.5 L of each gradient solution)] to give five fractions (fractions 2-1–2-5). An aliquot of fraction 2-3 (5.3 g, eluted with 70% MeOH from the MCI gel) was separated using a Sephadex LH-20 column (ϕ20 × 1000 mm; eluted with MeOH) and a μ-Bonda Pak C_18_ [ϕ25 × 200 mm; eluted with H_2_O-MeOH gradient [60%, 70%, 80%; each MeOH%; 135 mL of each gradient solution)], successively, and then purified with preparative HPLC [COSMOSIL AR-II (70% MeOH)] to give compounds **6** (4.5 mg). The third fraction (65.0 g, eluted by 100% MeOH) was further applied to a MCI gel CHP20P column [ϕ50 × 300 mm, eluted with H_2_O-MeOH gradient (0, 50, 60, 70, 80, 90, 100%; each MeOH%; 1.5 L of each gradient solution)] to give seven fractions (fractions. 3-1–3-7). An aliquot of fraction 3-4 (9.0 g, eluted with 70% MeOH from MCI gel) was loaded on a Sephadex LH-20 column (ϕ20 × 1000 mm, eluted with MeOH) and μ-Bonda Pak C_18_ [ϕ25 × 200 mm, eluted with H_2_O-MeOH gradient (60, 70, 80%; each MeOH%; 135 mL of each gradient solution)], and a SiO_2_ column [ϕ10 × 100 mm, eluted with CHCl_3__:_ MeOH: H_2_O = 9: 1: 0.1 (*v*/*v*)], successively, and then purified with preparative HPLC [Sunfire Prep C_18_ (70% MeOH)] to give compounds **1** (28.9 mg). An aliquot of fraction 3-5 (10.4 g, eluted by 80% MeOH from MCI gel) was subjected to a Sephadex LH-20 column (ϕ20 × 1000 mm, eluted with MeOH), and then purified with preparative HPLC [COSMOSIL 5C_18_ AR-II (70% MeOH)] to give compounds **5** (3.3 mg) and **2** (28.1 mg). An aliquot of fraction 3-6 (12.2 g, eluted by 90% MeOH from MCI gel) was subjected to a Sephadex LH-20 column (ϕ20 × 1000 mm, eluted with MeOH) and a μ-Bonda Pak C_18_ [ϕ25 × 200 mm, eluted with H_2_O-MeOH gradient (70, 80, 90%; each MeOH%; 135 mL of each gradient solution)], successively, and then purified with preparative HPLC [COSMOSIL 5C_18_ AR-II (85% MeOH)] to give compounds **3** (7.7 mg) and **4** (33.7 mg). Icaritin and desmethylicaritin were obtained by an enzymatic hydrolysis of glucosides **5** and **6**, respectively. To a solution of **5** (2.0 mg) in acetate buffer (1.0 mL, pH 5.0, 100 mM), β-glucosidase from almonds (5.0 mg, EC 3.2.1.21, Sigma) was added and incubated for 12 h at 37°C. The reaction was quenched by adding MeOH, and the solvent was evaporated *in vacuo* to get a residue. The residue was purified with a SiO_2_ column [ϕ8 × 40 mm, eluted with CHCl_3_: MeOH = 20: 1 (*v*/*v*)] to obtain the compound icaritin (1.0 mg). In the same manor described above, compound **6** (2.0 mg) was treated with β-glucosidase to obtain desmethylicaritin (1.0 mg). The chemical structure of compounds **1** (icariin), **2** (icariside II), **3** (limonianin), **4** (epimedokoreanin B), **5** (icariside I), **6** (epimedoside C), icaritin, and desmethylicaritin were identified by comparing with the authentication data (^1^H- and ^13^C-NMR data) published in literature (Mizuno et al., 1988; Li et al., 1994; Wen-Kui et al., 1998; Jung et al., 2005; Bacher et al., 2010; Quan et al., 2010).

### HPLC Analysis

HPLC profiling was analyzed on a SIMADZU LC-10AD pump, SIMADZU SPD-10A detector, SIMADZU CTO-10AC column heater, and column of COSMOSIL 5C_18_ AR-II (5 μm, ϕ4.6 × 250 mm, Nacalai Tasque Inc., Kyoto, Japan) with a flow rate of 1.0 mL/min, column temperature of 40°C, and detection wavelength of 280 nm. The stock solution of the compounds **1**–**6** were mixed with the same volume to make a standard solution. The solution was injected to the HPLC system (10 μL). The third fraction (MCI gel CHP-20P eluted by 100% MeOH) was dissolved in DMSO at 10 mg/mL concentration and injected to the HPLC system (10 μL).

### Preparation of Tumor Culture Supernatant (TCS)

Cell lines were maintained in culture medium for 24 h. The supernatant was used as the tumor culture supernatant (TCS) for the experiments.

### Measurement of the Effects of Extracts and Isolated Compounds on CD163 Expression

All the test samples were initially dissolved in DMSO (100 mg/mL for extracts and 10 mM for pure compounds). The DMSO solutions were then diluted with cell culture medium to a concentration of 100 μg/mL for extracts and 5–30 μM for pure compounds. In all the procedures, including extraction, elution of fractions, and isolation of compounds (icariin, icariside II, limonianin, epimedokoreanin B, icaritin, and desmethylicaritin), the mixtures were dissolved well to avoid any precipitates. HMDMs (1 × 10^4^ cells per well in a 96-well plate) were incubated with the extracts and isolated compounds from *Epimedii Herba* for 24 h along with IL-10, followed by the determination of CD163 expression using cell enzyme-linked immunosorbent assay (cell-ELISA) as described previously (Komohara et al., 2006). Briefly, each well of a 96-well plate was blocked with Block Ace (DS Pharma Biomedical, Osaka, Japan) and washed thrice with washing buffer (PBS containing 0.05% Tween 20). The wells were incubated with an anti-human CD163 antibody (AM-3K; 2 μg/mL) for 1 h. The wells were then incubated with a horseradish peroxidase (HRP)-conjugated anti-mouse IgG antibody after washing thrice with washing buffer, followed by addition of TMB Microwell Peroxidase Substrate (SeraCare Life Science Inc., Milford, MA, United States). The reaction was then terminated by the addition of 1 M sulfuric acid, and the absorbance was read at 450 nm using a micro-ELISA plate reader.

### Measurement of the Effects of the Isolated Compounds on IL-10, TNF-α, and IL-1β Secretion

Human monocyte-derived macrophages (1 × 10^4^ cells per well in a 96-well plate) were stimulated with 100 ng/mL LPS for 24 h after treatment with the compounds isolated from *Epimedii Herba* for 24 h in the presence of TCS. The secretion of IL-10, TNF-α, and IL-1β were measured using a cytokine ELISA kit (Thermo Fisher Scientific, Waltham, MA, United States).

### Measurement of the Effect of the Isolated Compounds on CD206 Expression

Human monocyte-derived macrophages (2 × 10^5^ cells per well in a 12-well plate) were incubated with the compounds isolated from *Epimedii Herba* for 24 h during incubation with IL-4, followed by the determination of CD206 expression by western blot analysis. The lyzed HMDMs were separated on a 10% sodium dodecyl sulfate (SDS)—polyacrylamide gel and transferred to a polyvinylidene fluoride (PVDF) transfer membrane (Millipore, Bedford, MA, United States). The membranes were incubated with an anti-human CD206 antibody (ab125028; Abcam, Cambridge, United Kingdom, 0.1 μg/mL), followed by visualization with an HRP-conjugated secondary anti-IgG antibody and the enhanced chemiluminescence (ECL) western blotting detection reagent (Thermo Fisher Scientific, Waltham, MA, United States). The membranes were reblotted with an anti-β-actin antibody as an internal calibration control.

### JAK/STAT Activation Assay

JAK1/STAT3 activation was determined by measuring the increased expression of phosphorylated STAT3 by western blot analysis as described previously (Fujiwara et al., 2011). Briefly, lyzed HMDMs and/or tumor cells were separated on a 10% SDS-polyacrylamide gel and transferred to a PVDF transfer membrane (Millipore, Bedford, MA, United States). The membranes were incubated with an anti-phosphorylated STAT3 antibody (D3A7; Cell Signaling Technology; 1:2000), an anti-phosphorylated JAK1 antibody (#3331; Cell Signaling Technology; 1:2000), and an anti-STAT3 antibody (sc-8019; Santa Cruz Biotechnology; 1:2000) (Yokogami et al., 2000; Duan and Simpson-Haidaris, 2006), followed visualization with an HRP-conjugated secondary anti-IgG antibody and the ECL western blotting detection reagent (Thermo Fisher Scientific, Waltham, MA, United States). The membranes were reblotted with an anti-β-actin antibody as an internal calibration control.

### Immunohistochemistry

Paraffin-embedded subcutaneous tumor tissues were sectioned (5 μM thick) and immunostained with pSTAT3, proliferating cell nuclear antigen (PCNA), Iba-1, CD3, CD34, and CD204. Anti-pSTAT3 (D3A7, Cell Signaling Technology; 1:200), anti-PCNA (M0879, DAKO; 1:200), anti-Iba-1 (019-19741, Wako; 1:4000), anti-CD3 (413591, Nichirei Biosciences; 1:2), anti-CD34 (ab8129, Abcam; 1:2000), and anti-CD204 (2F8, Invitrogen; 1:2000) antibodies were used as primary antibodies. The sections were subsequently treated with an HRP-conjugated secondary antibody (Nichirei, Tokyo, Japan), followed by the visualization with diaminobenzidine.

### Cell Proliferation and Cytotoxicity Assay

Briefly, 1 × 10^4^ tumor cells (Saos-2 and LM8) were cultured with the *Epimedii Herba*-derived compounds for 24 h in a 96-well plate. Cell viability was determined using the WST-8 assay (Dojin Chemical, Kumamoto, Japan) according to the manufacturer’s protocol.

### Co-culture and 5-Bromo-2′-Deoxyuridine (BrdU) Incorporation Assay

Tumor cells (1 × 10^4^ cells/well) and macrophages stimulated with or without the flavonoid compounds were directly co-cultured in 96-well plates for 2 days. 5-Bromo-2′-deoxyuridine (BrdU) incorporation was assayed using a BrdU Cell Proliferation Kit (Roche, Basel, Switzerland) according to the manufacturer’s protocol.

### Colony Formation Assay

Tumor cells (100 cells/well) were cultured in RPMI 1640 supplemented with 10% FBS and SphereMax (Wako) in a 96-well ultra-low attachment plate (Corning, NY, United States) for 10 days. The number of colonies (size > 100 μM) was counted under a microscope.

### Murine Sarcoma Model

Female C3H mice (8–10 weeks old) were purchased from CLEA Japan (Tokyo, Japan). LM8 cells (5 × 105 cells) were suspended in 100 μL DMEM culture medium and injected subcutaneously on back of the mice (Day 0). Vehicle (0.5% methylcellulose) or epimedokoreanin B (20 mg/kg) was administered orally thrice a week. The mice were sacrificed on day 17 and the subcutaneous tumor development and lung metastasis were analyzed. All animal experiments were approved by the Ethics Committee for Animal Experiments of Kumamoto University (Permission Number: A30-047) and were performed in accordance with the Guidelines for Laboratory Animal Experiments.

### Statistical Analysis

All data are representative of two or three independent experiments. Data are expressed as the mean ± standard deviation (SD). Differences between groups were examined using the Mann–Whitney *U*-test and the non-repeated measures ANOVA to determine the statistical significance. A *p*-value < 0.05 was considered to be a statistically significant difference.

## Results

### Effects of the Crude Extract and Isolated Compounds From *Epimedii Herba* on Macrophage Activation

**FIGURE 1:**
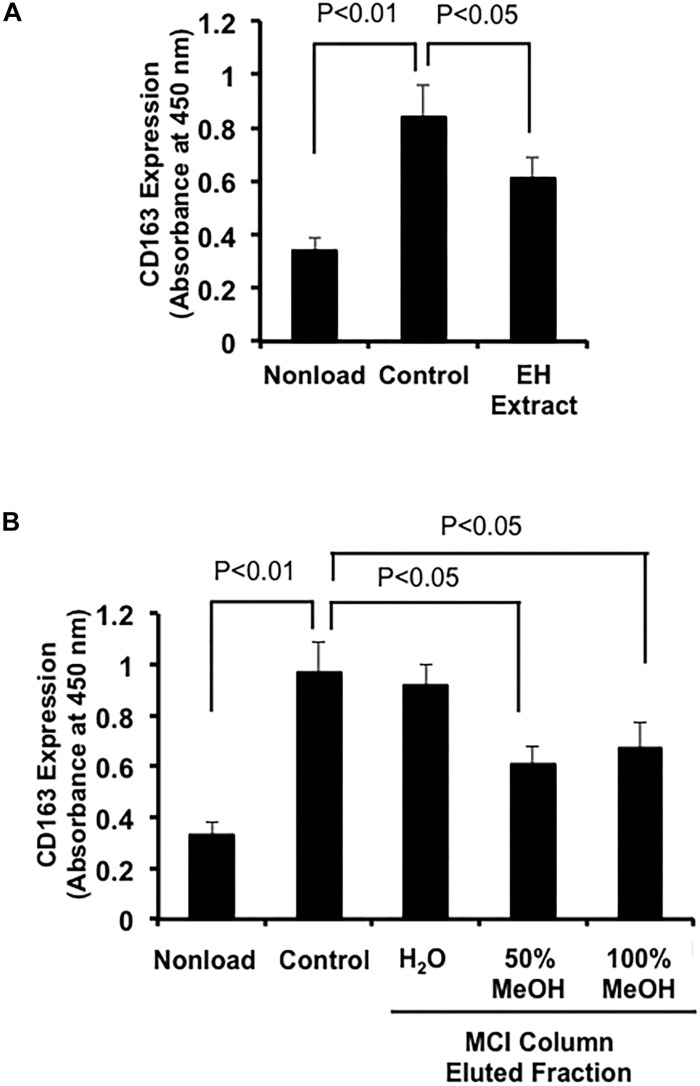
Effects of extract prepared from *Epimedii Herba* on IL-10-induced CD163 expression in human macrophages. **(A)** HMDMs (5 × 10^4^ cells per well in 96-well plates) were incubated with *Epimedii Herba* extract (200 μg/mL) in the presence of IL-10 (20 ng/mL) for 24 h, followed by the determination of CD163 expression using cell-ELISA. **(B)** HMDMs (5 × 10^4^ cells per well in 96-well plates) were incubated with H_2_O-eluted and MeOH-eluted fractions prepared from the *Epimedii Herba* extract (200 μg/mL) in the presence of IL-10 (20 ng/mL) for 24 h. CD163 expression was then determined using cell-ELISA. The data are presented as mean ± SD.

**FIGURE 2:**
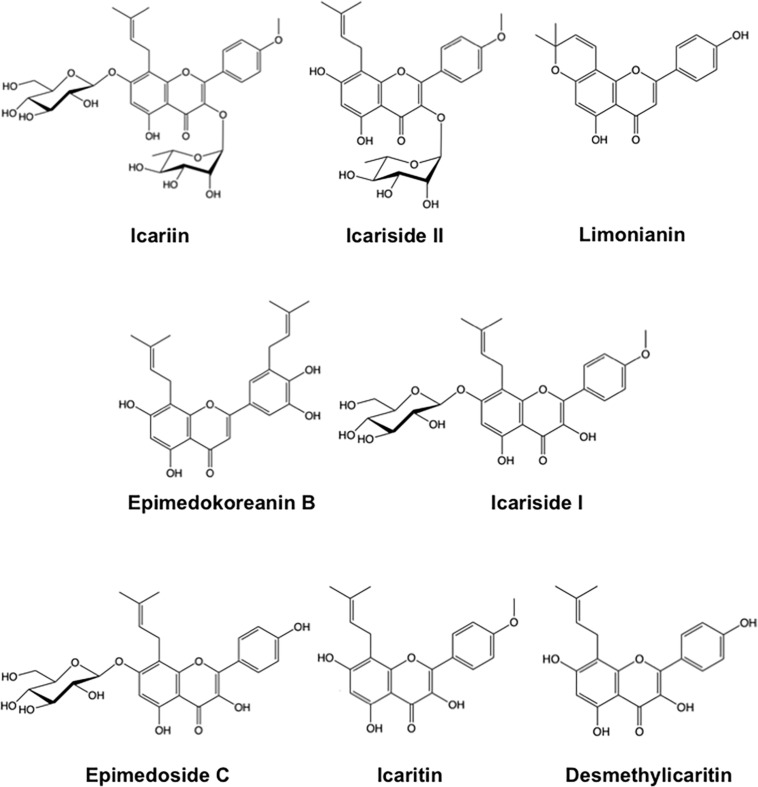
Chemical structure of compounds derived from *Epimedii Herba.*

**FIGURE 3:**
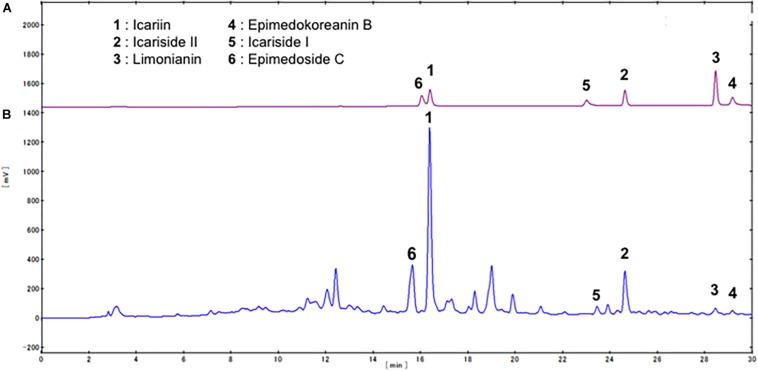
HPLC profile of compounds contained in *Epimedii Herba.* **(A)** HPLC analysis of the standard compounds isolated from *Epimedii Herba*. **(B)** HPLC profile of the fraction prepared using MCI gel CHP-20P (eluted with MeOH).

We first examined the effects of a crude extract prepared from *Epimedii Herba* on IL-10-induced expression of CD163, an M2 phenotype marker, in HMDMs by cell-ELISA. The *Epimedii Herba* extract significantly inhibited IL-10-induced CD163 expression (Figure 1A). Next, the *Epimedii Herba* extract was subjected to MCI column chromatography and eluted with H_2_O and 50–100% MeOH to obtain three fractions. As shown in Figure 1B, both the 50% MeOH fraction and the 100% MeOH fraction significantly suppressed the CD163 expression, suggesting that these fractions contained compound(s) that could inhibit CD163 expression. Therefore, we isolated the compounds from these fractions and identified them as icariin, icariside II, limonianin, epimedokoreanin B, icariside I, and epimedoside C (Figure 2). Icariin, a flavonoid glycoside, is the main constituent in the MeOH-eluted fractions, as shown in Figures 3A,B. Because the sugar chains of glycosides such as icariin, icariside II, icariside I, and epimedoside C are degraded by the action of intestinal bacteria after oral administration, the aglycons such as icaritin and desmethylicaritin (Figure 2) can be assumed to act as the physiologically active compounds (Kida et al., 1997; Hasegawa and Uchiyama, 1998).

**FIGURE 4:**
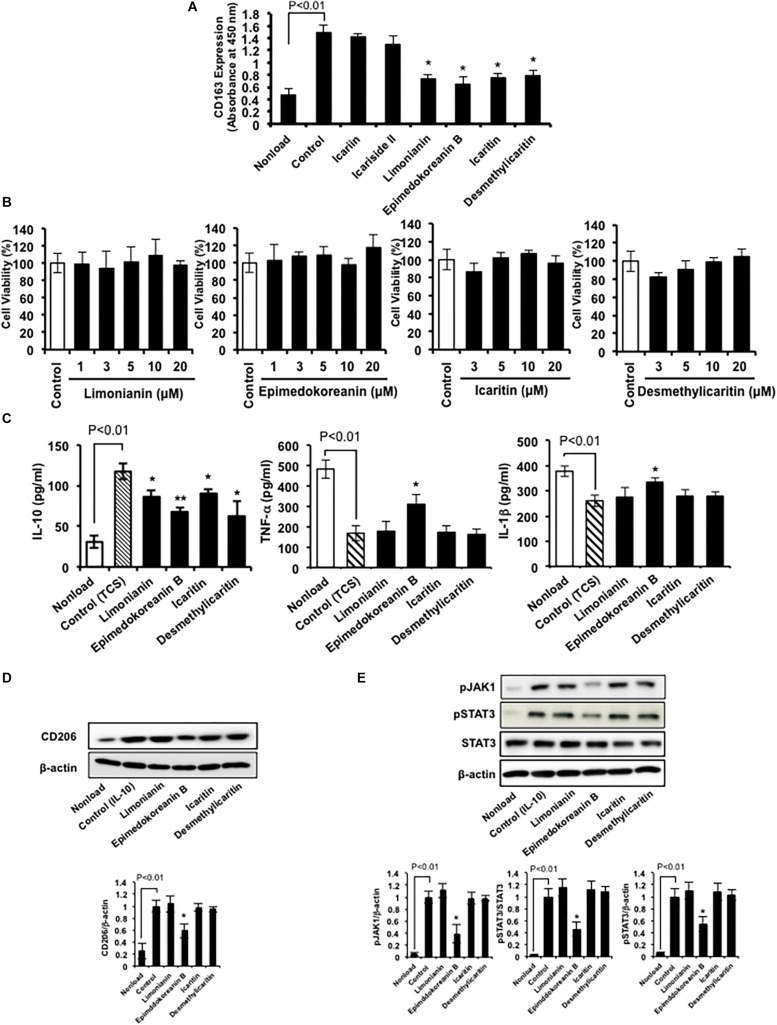
Effects of compounds isolated from *Epimedii Herba* on macrophage activation. **(A)** HMDMs were incubated with compounds (5 μM) in the presence of IL-10 (20 ng/mL) for 24 h, followed by measurement of CD163 expression using cell-ELISA. **(B)** HMDMs were incubated with the indicated concentrations of the four flavonoid compounds for 24 h, followed by the determination of cell viability using WST-8 assay. **(C)** HMDMs were incubated with the four flavonoid compounds (5 μM) for 24 h in the presence of tumor culture supernatant (TCS), followed by the determination of IL-10, TNF-α, and IL-1β secretion by ELISA. **(D)** HMDMs were incubated with flavonoid compounds (5 μM) in the presence of IL-4 (20 nM) for 24 h, followed by the determination of CD206 and β-actin protein expression by western blot analysis. **(E)** HMDMs were incubated with the four flavonoid compounds (5 μM) in the presence of IL-10 (20 nM) for 6 h, followed by the determination of phosphorylated JAK1, phosphorylated STAT3, STAT3, and β-actin protein expression by western blot analysis. Experiments ware repeated three times with almost identical results. The data are presented as mean ± SD. *p < 0.05, **p < 0.01 vs. control.

Therefore, we examined the effects of these compounds (Figure 2) on CD163 expression in HMDMs. We found that limonianin, epimedokoreanin B, icaritin, and desmethylicaritin inhibited IL-10-induced CD163 expression (Figure 4A), while they had no cytotoxic effect on the macrophages (Figure 4B), suggesting that aglycons are the active compounds in this study. Therefore, we chose to use limonianin, epimedokoreanin B, icaritin, and desmethylicaritin for further evaluations.

Next, we measured the effect of limonianin, epimedokoreanin B, icaritin, and desmethylicaritin on the secretion of IL-10, TNF-α, and IL-1β from HMDMs induced by TCS stimulation. TCS stimulation increased IL-10 secretion, a cytokine marker of M2 macrophages, and decreased TNF-α and IL-1β secretion, cytokine markers of M1 macrophages, in HMDMs (Figure 4C). Under the assay conditions employed, the tested compounds significantly inhibited TCS-induced IL-10 secretion (Figure 4C). In addition, epimedokoreanin B also enhanced TNF-α and IL-1β secretion reduced by TCS stimulation (Figure 4C). Furthermore, epimedokoreanin B also inhibited IL-4-induced CD206 expression, another M2 phenotype marker (Figure 4D). These data indicate that epimedokoreanin B has a potentially inhibitory effect on M2 polarization of HMDMs.

### Effect of Limonianin, Epimedokoreanin B, Icaritin, and Desmethylicaritin on STAT3 Activation in Macrophages

Since the activation of STAT3 contributes to the M2 polarization of macrophages (Mantovani et al., 2002), we next examined the effect of limonianin, epimedokoreanin B, icaritin, and desmethylicaritin on IL-10-induced JAK1/STAT3 activation in HMDMs. As shown in Figure 4E, IL-10 induced JAK1/STAT3 activation in HMDMs. Under the assay conditions employed, epimedokoreanin B significantly suppressed IL-10-induced JAK1/STAT3 activation. These results suggest that epimedokoreanin B regulates conversion of M2 into M1 phenotype in HMDMs by inhibiting STAT3 activation.

### Effect of Limonianin, Epimedokoreanin B, Icaritin, and Desmethylicaritin on STAT3 Activation and Proliferation in Tumor Cells

**FIGURE 5:**
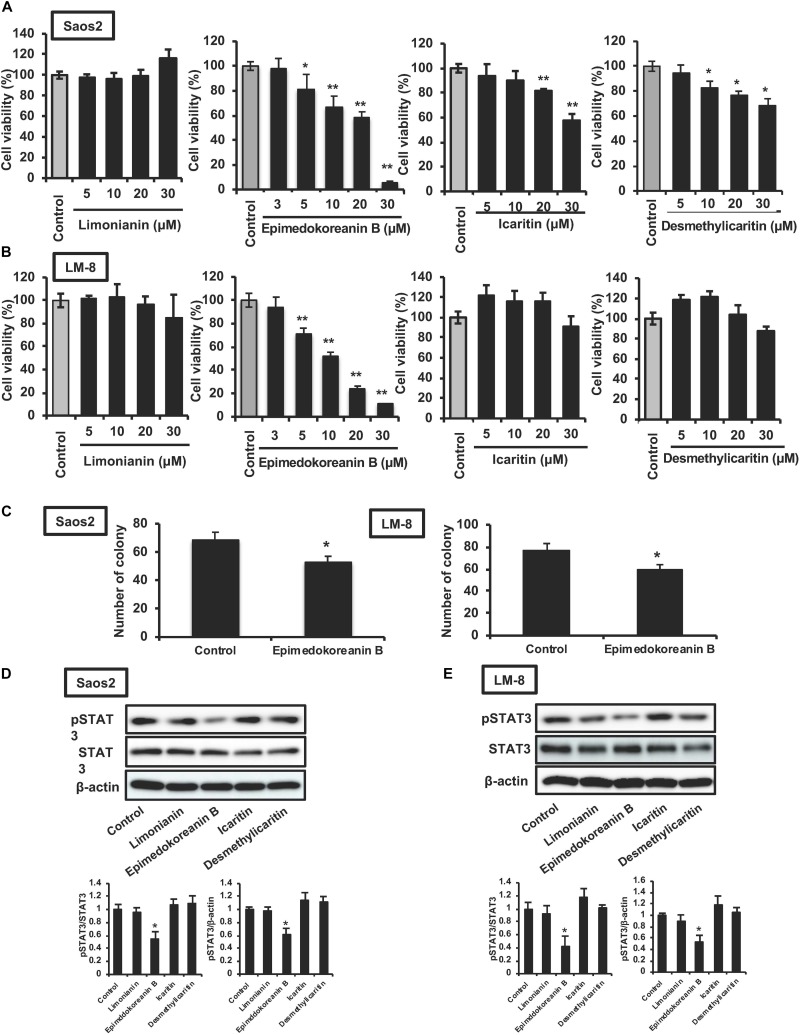
Effects of compounds isolated from *Epimedii Herba* on STAT3 activation and proliferation in tumor cells. Saos-2 human osteosarcoma cells **(A)** and LM8 mouse sarcoma cells **(B)** were incubated with the indicated concentrations of the four flavonoid compounds for 24 h, followed by determination of cell viability using the WST-8 assay. **(C)** Tumor cells were incubated with epimedokoreanin B (5 μM) for 10 days in the presence of SphereMax, followed by determination of the number of colonies under a microscope. Saos-2 human osteosarcoma cells **(D)** and LM8 mouse sarcoma cells **(E)** were incubated with flavonoid compounds (10 μM) for 24 h. The expression of phosphorylated STAT3, STAT3, and β-actin was determined by western blot analysis. Experiments ware repeated three times with almost identical results. The data are presented as mean ± SD. *p < 0.01, **p < 0.005 vs. control.

It is well known that the activation of STAT3 is critically associated with tumorigenesis (Thoennissen et al., 2009), and STAT3 is considered an important target for anticancer therapy (Iwamaru et al., 2007; Fuh et al., 2009). Therefore, we investigated the effect of limonianin, epimedokoreanin B, icaritin, and desmethylicaritin on the proliferation of Saos-2 human osteosarcoma cells and LM8 mouse sarcoma cells. As shown in Figure 5A, epimedokoreanin B, icaritin, and desmethylicaritin significantly inhibited the proliferation of Saos-2 cells. Among these compounds, epimedokoreanin B had a strong inhibitory effect on Saos-2 cell proliferation. Epimedokoreanin B also significantly inhibited the proliferation of LM8 cells (Figure 5B). Furthermore, epimedokoreanin B also inhibited colony formation in both Saos-2 and LM-8 cells (Figure 5C). As shown in Figures 5D,E, STAT3 was constantly activated in both cell lines. Under the assay conditions used, epimedokoreanin B also suppressed STAT3 activation in both Saos-2 cells and LM8 cells (Figures 5D,E). These data suggest that inhibition of STAT3-related signal pathway by epimedokoreanin B suppresses tumor cell proliferation.

### Effect of Epimedokoreanin B on Tumor Proliferation Under Co-culture Conditions With Macrophages and Sarcoma Cells

**FIGURE 6:**
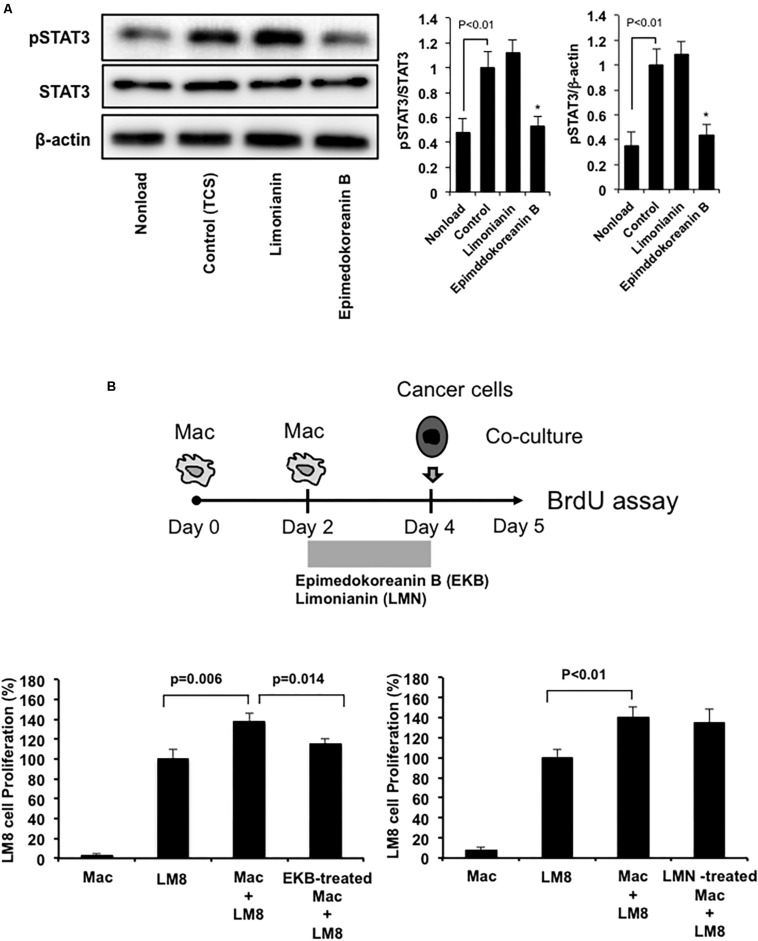
Effect of epimedokoreanin B on cell-cell interactions between mouse macrophages and mouse sarcoma cell line. **(A)** Mouse peritoneal macrophages were incubated with epimedokoreanin B (5 μM) and limonianin (5 μM) in the presence of TCS from LM8 cells for 6 h. The expression of phosphorylated STAT3, STAT3, and β-actin was determined by western blot analysis. **(B)** Mouse peritoneal macrophages were incubated with LM8 sarcoma cells under direct co-culture conditions for 24 h after stimulation with epimedokoreanin B (3 μM) and limonianin (3 μM) for 2 days, followed by the determination of proliferation by the BrdU incorporation assay. Experiments ware repeated three times with almost identical results. The data are presented as mean ± SD. *p < 0.05 vs. control.

Epimedokoreanin B also suppressed TCS-induced STAT3 activation in mouse peritoneal macrophages (Figure 6A). On the other hand, limonianin had no effect on TCS-induced STAT3 activation (Figure 6A). These data suggest that epimedokoreanin B regulates macrophage activation in both human and mouse macrophages. We next tested whether the macrophages treated with the flavonoid compounds (epimedokoreanin B and limonianin) inhibit sarcoma cell proliferation in a co-culture study using mouse macrophages and the LM8 mouse sarcoma cell line. Mouse peritoneal macrophages and mouse LM8 sarcoma cells were co-cultured, and the BrdU incorporation assay was performed, as shown in Figure 6B. Co-culture with macrophages enhanced tumor cell proliferation (Figure 6B). This enhanced effect was reduced by co-culture with epimedokoreanin B-treated macrophages (Figure 6B), indicating that epimedokoreanin B suppresses tumor proliferation by regulating macrophage activation.

### Epimedokoreanin B Suppressed Subcutaneous Tumor Development

**FIGURE 7:**
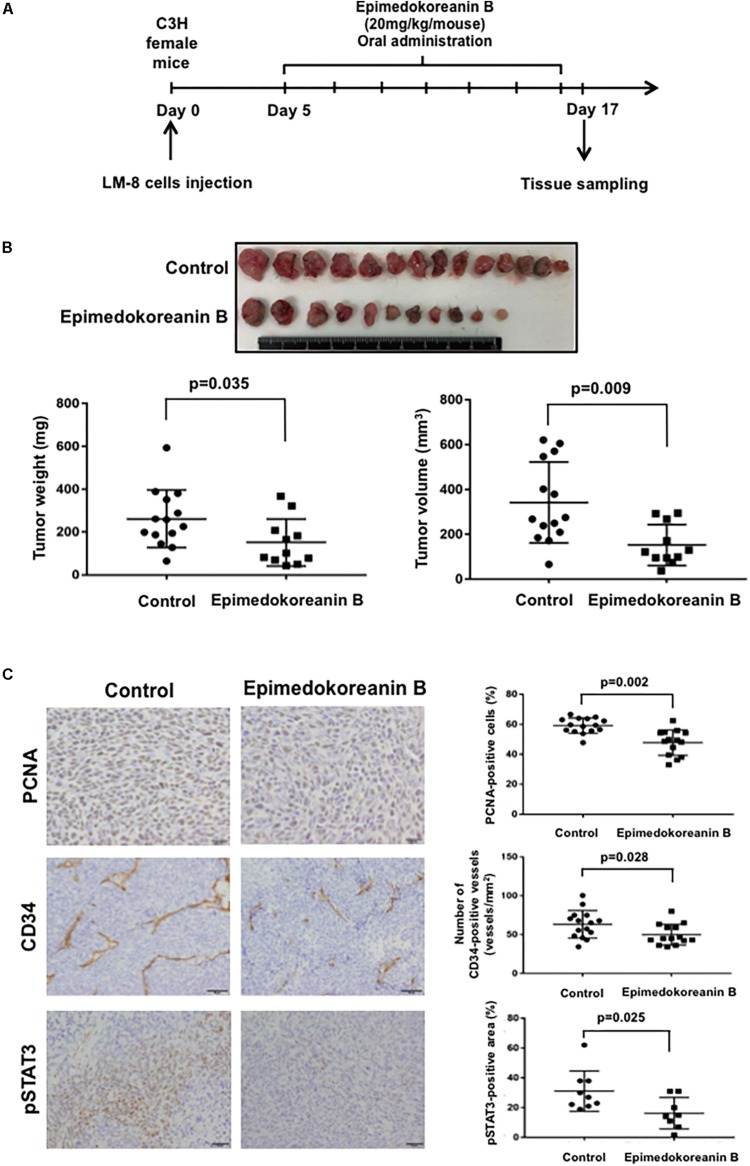
Effect of epimedokoreanin B on tumor progression in LM8 cell-injected mice. Epimedokoreanin B (20 mg/kg) was administered to mice injected with LM8 cells as described in the procedure **(A)**, followed by the determination of subcutaneous tumor weights **(B)**. PCNA expression, CD34 expression, and STAT3 activation in subcutaneous tumor tissues were evaluated by immunostaining **(C)**.

**FIGURE 8:**
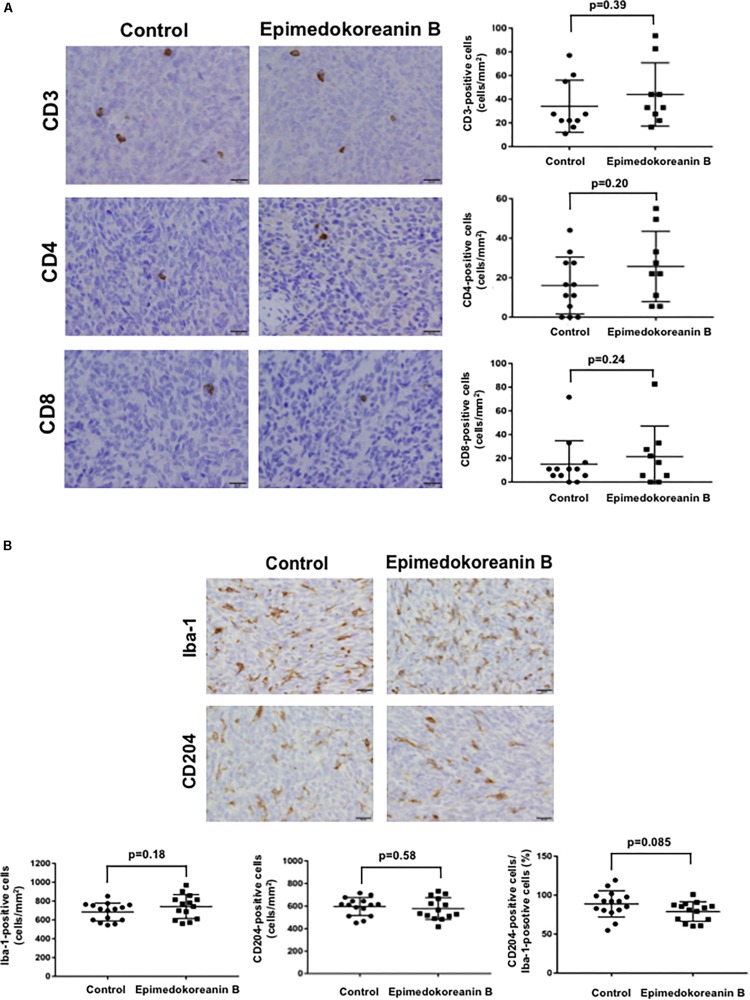
Number of lymphocytes and macrophages in tumor tissue. **(A)** CD3^+^, CD4^+^, and CD8^+^ lymphocytes in subcutaneous tumor tissues evaluated by immunostaining. **(B)** Iba-1- and CD204-positive macrophages in subcutaneous tumor tissues were evaluated by immunostaining.

To verify the antitumor effects of epimedokoreanin B in an *in vivo* study, we next investigated the effects of epimedokoreanin B in a mouse tumor model. Epimedokoreanin B was administered orally after the subcutaneous injection of LM8 cells in C3H mice, as shown in Figure 7A. On day 17 following tumor injection, subcutaneous tumors (>10 mm) were detected in all the control mice. On the other hand, epimedokoreanin B significantly inhibited subcutaneous tumor development (Figure 7B). We next performed immunohistochemical studies to examine the effects of epimedokoreanin B on the subcutaneous tumor cells. Tumor cell proliferation was evaluated by immunostaining for PCNA, and angiogenesis was evaluated by immunostaining for CD34. Both PCNA and CD34 expression was reduced following treatment with epimedokoreanin B (Figure 7C). STAT3 activation in the subcutaneous tumor tissue was also reduced following epimedokoreanin B administration (Figure 7C). However, the numbers of Iba-1-positive macrophages (pan-macrophages), CD204-positive macrophages (M2-like macrophages), CD3-positive lymphocytes, CD4-positive lymphocytes, and CD8-positive lymphocytes in subcutaneous tumor tissue remained unchanged following epimedokoreanin B administration (Figures 8A,B). However, the ratio of CD204-positive cells to Iba-1-positive cells decreased in response to epimedokoreanin B administration. These data indicate that epimedokoreanin B also inhibits tumor development in a mouse model by suppressing STAT3 activation.

## Discussion

Flavonoid compounds from *Epimedii Herba* have been shown to have various activities. Desmethylicaritin has an inhibitory effect on LPS-induced NO production in murine macrophages by suppressing iNOS activity (Chen et al., 2008) and an inhibitory effect on tumor proliferation in U87MG human glioblastoma cells (Kang et al., 2016). In the present study, desmethylicaritin inhibited the proliferation of Saos-2 cells (Figure 5A). It has been reported that icaritin has anti-inflammatory effects on mouse peritoneal macrophages by suppressing both p38 and JNK activation (Lai et al., 2013) and that it exerts antitumor and anti-inflammatory effects by modulating the function of myeloid-derived suppressive cells (MDSCs) (Zhou et al., 2011). However, there are limited number of reports on the biological activities of limonianin and epimedokoreanin B. We believe that this is the first report describing the antitumor effect of epimedokoreanin B both *in vitro* and *in vivo*.

In the present study, CD163 was used to evaluate M2 polarization in HMDMs. CD163 is a well-documented marker for detecting M2-like macrophages in paraffin-embedded surgical specimens (Komohara et al., 2006). In clinicopathological studies of malignant tumors using CD163 to detect M2-like TAMs, patients with glioma (Komohara et al., 2011b), follicular lymphoma (Clear et al., 2010), renal cancer (Komohara et al., 2011a), and pancreatic cancer (Kurahara et al., 2011) were shown to have poorer clinical prognosis in the presence of TAMs with a higher expression of CD163. CD163 is a member of the scavenger receptor cysteine-rich protein superfamily. It binds the hemoglobin–haptoglobin (Hb-Hp) complex (Kristiansen et al., 2001) and subsequently induces IL-10 secretion and HO-1 expression (Philippidis et al., 2003), which suggests that CD163 contributes to immunosuppression. Therefore, inhibition of TAM polarization to a CD163-positive M2 phenotype is a probable therapeutic strategy for cancer. In our previous studies, several compounds that suppress the polarization of macrophages to the CD163-positive M2 phenotype were discovered using an established screening system based on CD163-specific cell-ELISA. Corosolic acid and onionin A inhibit tumor proliferation by suppressing M2 polarization of macrophages as shown in *in vitro* and *in vivo* studies (Fujiwara et al., 2011, 2016; Horlad et al., 2013; Tsuboki et al., 2016). We also recently reported that the expression of CD163 on macrophages is associated with poor prognosis in undifferentiated human pleomorphic sarcoma. CD163 promotes murine sarcoma progression by inducing IL-6 secretion (Shiraishi et al., 2018), which indicates the pro-tumorigenic role of TAMs expressing CD163 in sarcoma. In the present study, we demonstrated that four flavonoid compounds isolated from *Epimedii Herba* inhibited M2 marker expression (CD163 and IL-10) in HMDMs (Figures 4A,C), while having no cytotoxic effect on these cells (Figure 4B).

We also demonstrated that limonianin and epimedokoreanin B inhibited IL-10-induced STAT3 activation in HMDMs (Figure 4D). It is known that the excessive activation of STAT3 in tumor cells is associated with poor prognosis, and IL-6, a classic STAT3-activator produced by mesenchymal stem cells, induces STAT3 activation in tumor cells such as Saos-2 osteosarcoma cells and consequently promotes tumor proliferation and metastasis (Tu et al., 2012). It was recently reported that CD163-positive macrophages are also associated with both proliferation in Saos-2 cells and tumor progression in a sarcoma-bearing mouse model by IL-6-induced STAT3 activation (Shiraishi et al., 2018). Epimedokoreanin B showed an inhibitory effect on STAT3 activation in both macrophages and tumor cells (Figures 4D, 5C,D), and epimedokoreanin B suppressed tumor progression in the LM8 tumor-bearing mouse model in the present study (Figure 7B). Furthermore, administration of epimedokoreanin B significantly decreased both pSTAT3-positive area and PCNA-positive cells in subcutaneous tumor tissues (Figure 7C), thus indicating that epimedokoreanin B suppresses tumor development in a mouse model by inhibiting STAT3 activation in both macrophages and tumor cells. STAT3 is a known M2 phenotype-inducer in macrophages. As shown in Figure 8, epimedokoreanin B reduced the ratio of CD204^+^ M2 macrophages to Iba-1^+^ total macrophages in the subcutaneous tumor tissues. However, there was no significant effect on M2-polarization in the *in vivo* mouse model in the present study. The reason for this discrepancy may be the differences in sensitivity of humans and mice to these compounds. Since lack of M2-related genes such as that coding CD163 was seen in mouse TAMs (Shiraishi et al., 2018), the differences in the gene expression profiles between humans and mice may also cause this discrepancy.

In several human tumors, a STAT3 inhibitor rescued the expression of proinflammatory cytokines and costimulatory molecules on TAMs and peripheral macrophages and resulted in the enhancement of immune responses (Hussain et al., 2007), thus indicating the significance of STAT3 activation in cell–cell interactions between TAMs and tumor cells. STAT3 is involved not only in macrophage differentiation but also in tumor cell proliferation (Yu et al., 2007). Activation of STAT3 in glioma cells is closely correlated with poor clinical prognoses in patients with grade III glioma (Abou-Ghazal et al., 2008). Therefore, STAT3 is considered to be a target molecule in several tumor types (Brantley and Benveniste, 2008). Furthermore, the activation of STAT3 in tumor cells was reported to cause resistance to anticancer therapies, such as radiotherapy and chemotherapy (Gao et al., 2010).

Niclosamide, a selective STAT3 inhibitor, had a stronger inhibitory effect on STAT3 activation compared to epimedokoreanin B (Supplementary Figure S1). However, niclosamide affected the vitality of human macrophages, thus suggesting that direct STAT3 targeting agents may have undesirable side effect on macrophages (Supplementary Figure S1). It is known that natural compound-derived STAT3 inhibitors such as curcumin and butein are non-specific and target STAT3 indirectly. We believe that epimedokoreanin B also targets STAT3 indirectly.

We also previously revealed that natural compound-derived STAT3 inhibitors, such as corosolic acid and onionin A, enhanced the efficacy of chemotherapeutic agents against tumor cells (Fujiwara et al., 2013, 2014). In the present study, we revealed that epimedokoreanin B significantly inhibited STAT3 activation in human macrophages and tumor cells and suppressed macrophage polarization to the M2 phenotype. In addition, the oral administration of epimedokoreanin B significantly suppressed tumor development in tumor-bearing mice.

These findings indicate that epimedokoreanin B is a functional compound for antitumor therapy that regulates macrophage activation and tumor proliferation. Epimedokoreanin B may also directly suppress tumor proliferation and enhance tumor sensitivity to radiotherapy and chemotherapy. Therefore, epimedokoreanin B may be a useful compound for anticancer therapy, and the products derived from *Epimedii Herba* may be useful for tumor prevention.

## Data Availability Statement

All datasets generated for this study are included in the article/Supplementary Material.

## Ethics Statement

All protocols using human materials were approved by the Kumamoto University Review Board (No. 486) and were conducted in accordance with the approved guidelines. All animal experiments were approved by the Ethics Committee for Animal Experiments of Kumamoto University (Permission Number: A30-047) and were performed in accordance with the Guidelines for Laboratory Animal Experiments.

## Author Contributions

YF and YK designed the experiments. CP, YF, HH, DS, TIr, JT, and TIk analyzed the data. CP, YF, and YK wrote the manuscript. CP, YF, HH, and DS performed the experiments. All authors critically reviewed and approved the final form of the manuscript.

## Conflict of Interest

The authors declare that the research was conducted in the absence of any commercial or financial relationships that could be construed as a potential conflict of interest.

## References

[B1] Abou-Ghazal M. Yang D. S. Qiao W. Reina-Ortiz C. Wei J. Kong L. Y. (2008). The incidence, correlation with tumor-infiltrating inflammation, and prognosis of phosphorylated STAT3 expression in human gliomas. *Clin. Cancer Res.* 14 8228–8235. 10.1158/1078-0432.CCR-08-1329 19088040 PMC2605668

[B2] Bacher M. Brader G. Greger H. Hofer O. (2010). Complete 1H and 13C NMR data assignment of new constituents from *Severinia buxifolia*. *Magn. Reson. Chem.* 48 83–88. 10.1002/mrc.2548 19937908

[B3] Brantley E. C. Benveniste E. N. (2008). Signal transducer and activator of transcription-3: a molecular hub for signaling pathways in gliomas. *Mol. Cancer Res.* 6 675–684. 10.1158/1541-7786.MCR-07-2180 18505913 PMC3886801

[B4] Chen C. C. Tsai P. C. Wei B. L. Chiou W. F. (2008). 8-Prenylkaempferol suppresses inducible nitric oxide synthase expression through interfering with JNK-mediated AP-1 pathway in murine macrophages. *Eur. J. Pharmacol.* 590 430–436. 10.1016/j.ejphar.2008.05.018 18579129

[B5] Clear A. J. Lee A. M. Calaminici M. Ramsay A. G. Morris K. J. Hallam S. (2010). Increased angiogenic sprouting in poor prognosis FL is associated with elevated numbers of CD163+ macrophages within the immediate sprouting microenvironment. *Blood* 115 5053–5056. 10.1182/blood-2009-11-253260 20375314 PMC2890144

[B6] Duan H. O. Simpson-Haidaris P. J. (2006). Cell type-specific differential induction of the human gamma-fibrinogen promoter by interleukin-6. *J. Biol. Chem.* 281 12451–12457. 10.1074/jbc.M600294200 16524883

[B7] Espinosa I. Beck A. H. Lee C. H. Zhu S. Montgomery K. D. Marinelli R. J. (2009). Coordinate expression of colony-stimulating factor-1 and colony-stimulating factor-1-related proteins is associated with poor prognosis in gynecological and nongynecological leiomyosarcoma. *Am. J. Pathol.* 174 2347–2356. 10.2353/ajpath.2009.081037 19443701 PMC2684198

[B8] Fuh B. Sobo M. Cen L. Josiah D. Hutzen B. Cisek K. (2009). LLL-3 inhibits STAT3 activity, suppresses glioblastoma cell growth and prolongs survival in a mouse glioblastoma model. *Br. J. Cancer* 100 106–112. 10.1038/sj.bjc.6604793 19127268 PMC2634692

[B9] Fujiwara Y. Horlad H. Shiraishi D. Tsuboki J. Kudo R. Ikeda T. (2016). Onionin A, a sulfur-containing compound isolated from onions, impairs tumor development and lung metastasis by inhibiting the protumoral and immunosuppressive functions of myeloid cells. *Mol. Nutr. Food Res.* 60 2467–2480. 10.1002/mnfr.201500995 27393711

[B10] Fujiwara Y. Komohara Y. Ikeda T. Takeya M. (2011). Corosolic acid inhibits glioblastoma cell proliferation by suppressing the activation of signal transducer and activator of transcription-3 and nuclear factor-kappa B in tumor cells and tumor-associated macrophages. *Cancer Sci.* 102 206–211. 10.1111/j.1349-7006.2010.01772.x 21073634 PMC11158718

[B11] Fujiwara Y. Takaishi K. Nakao J. Ikeda T. Katabuchi H. Takeya M. (2013). Corosolic acid enhances the antitumor effects of chemotherapy on epithelial ovarian cancer by inhibiting signal transducer and activator of transcription 3 signaling. *Oncol. Lett.* 6 1619–1623. 10.3892/ol.2013.1591 24260055 PMC3834045

[B12] Fujiwara Y. Takeya M. Komohara Y. (2014). A novel strategy for inducing the antitumor effects of triterpenoid compounds: blocking the protumoral functions of tumor-associated macrophages via STAT3 inhibition. *Biomed. Res. Int.* 2014:348539. 10.1155/2014/348539 24738052 PMC3967493

[B13] Gao L. Li F. Dong B. Zhang J. Rao Y. Cong Y. (2010). Inhibition of STAT3 and ErbB2 suppresses tumor growth, enhances radiosensitivity, and induces mitochondria-dependent apoptosis in glioma cells. *Int. J. Radiat. Oncol. Biol. Phys.* 77 1223–1231. 10.1016/j.ijrobp.2009.12.036 20610043

[B14] Hasegawa H. Uchiyama M. (1998). Antimetastatic efficacy of orally administered ginsenoside Rb1 in dependence on intestinal bacterial hydrolyzing potential and significance of treatment with an active bacterial metabolite. *Planta Medica* 64 696–700. 10.1055/s-2006-957560 9933987

[B15] Horlad H. Fujiwara Y. Takemura K. Ohnishi K. Ikeda T. Tsukamoto H. (2013). Corosolic acid impairs tumor development and lung metastasis by inhibiting the immunosuppressive activity of myeloid-derived suppressor cells. *Mol. Nutr. Food Res.* 57 1046–1054. 10.1002/mnfr.201200610 23417831

[B16] Hussain S. F. Kong L. Y. Jordan J. Conrad C. Madden T. Fokt I. (2007). A novel small molecule inhibitor of signal transducers and activators of transcription 3 reverses immune tolerance in malignant glioma patients. *Cancer Res.* 67 9630–9636. 10.1158/0008-5472.CAN-07-1243 17942891

[B17] Iwamaru A. Szymanski S. Iwado E. Aoki H. Yokoyama T. Fokt I. (2007). A novel inhibitor of the STAT3 pathway induces apoptosis in malignant glioma cells both in vitro and in vivo. *Oncogene* 26 2435–2444. 10.1038/sj.onc.1210031 17043651

[B18] Jung H. J. Kang S. S. Hyun S. K. Choi J. S. (2005). In vitro free radical and ONOO- scavengers from *Sophora flavescens*. *Arch. Pharm. Res.* 28 534–540. 10.1007/BF02977754 15974438

[B19] Kang C. W. Kim N. H. Jung H. A. Choi H. W. Kang M. J. Choi J. S. (2016). Desmethylanhydroicaritin isolated from *Sophora flavescens*, shows antitumor activities in U87 MG cells via inhibiting the proliferation, migration and invasion. *Environ. Toxicol. Pharmacol.* 43 140–148. 10.1016/j.etap.2016.03.003 26991848

[B20] Kida H. Akao T. Meselhy M. R. Hattori M. (1997). Enzymes responsible for the metabolism of saikosaponins from *Eubacterium* sp. A-44, a human intestinal anaerobe. *Biol. Pharm. Bull.* 20 1274–1278. 10.1248/bpb.20.1274 9448103

[B21] Komohara Y. Hirahara J. Horikawa T. Kawamura K. Kiyota E. Sakashita N. (2006). AM-3K, an anti-macrophage antibody, recognizes CD163, a molecule associated with an anti-inflammatory macrophage phenotype. *J. Histochem. Cytochem.* 54 763–771. 10.1369/jhc.5A6871.2006 16517975

[B22] Komohara Y. Horlad H. Ohnishi K. Fujiwara Y. Suzu S. Eto M. (2011a). Macrophage infiltration and its prognostic relevance in clear cell renal cell carcinoma. *Cancer Sci.* 102 1424–1431. 10.1111/j.1349-7006.2011.01945.x 21453387

[B23] Komohara Y. Jinushi M. Takeya M. (2014). Clinical significance of macrophage heterogeneity in human malignant tumors. *Cancer Sci.* 105 1–8. 10.1111/cas.12314 24168081 PMC4317877

[B24] Komohara Y. Niino D. Ohnishi K. Ohshima K. Takeya M. (2015). Role of tumor-associated macrophages in hematological malignancies: TAMs in hematological malignancies. *Pathol. Int.* 65 170–176. 10.1111/pin.12259 25707506

[B25] Komohara Y. Ohnishi K. Kuratsu J. Takeya M. (2011b). Possible involvement of the M2 anti-inflammatory macrophage phenotype in growth of human gliomas. *J. Pathol.* 216 15–24. 10.1002/path.2370 18553315

[B26] Kristiansen M. Graversen J. H. Jacobsen C. Sonne O. Hoffman H. J. Law S. K. (2001). Identification of the haemoglobin scavenger receptor. *Nature* 409 198–201. 10.1038/35051594 11196644

[B27] Kurahara H. Shinchi H. Mataki Y. Maemura K. Noma H. Kubo F. (2011). Significance of M2-polarized tumor-associated macrophage in pancreatic cancer. *J. Surg. Res.* 167 e211–e219. 10.1016/j.jss.2009.05.026 19765725

[B28] Lai X. Ye H. Sun C. Huang X. Tang X. Zeng X. (2013). Icaritin exhibits anti-inflammatory effects in the mouse peritoneal macrophages and peritonitis model. *Int. Immunopharmacol.* 16 41–49. 10.1016/j.intimp.2013.03.025 23566810

[B29] Lewis C. E. Pollard J. W. (2006). Distinct role of macrophages in different tumor microenvironments. *Cancer Res.* 66 605–612. 10.1158/0008-5472.CAN-05-4005 16423985

[B30] Li W. K. Zhang R. Y. Xiao P. G. (1994). Epimedokoreanin B and Epimedokoreanin C from the aerial parts of *Epimedium koreanum* Nakai. *Acta Pharm. Sin.* 29 835–839.

[B31] Mantovani A. Biswas S. K. Galdiero M. R. Sica A. Locati M. (2013). Macrophage plasticity and polarization in tissue repair and remodeling: macrophage plasticity and polarization in tissue repair and remodeling. *J. Pathol.* 229 176–185. 10.1016/j.it.2004.09.015 23096265

[B32] Mantovani A. Sica A. Sozzani S. Allavena P. Vecchi A. Locati M. (2004). The chemokine system in diverse forms of macrophage activation and polarization. *Trends Immunol.* 25 677–686. 10.1016/j.it.2004.09.015 15530839

[B33] Mantovani A. Sozzani S. Locati M. Allavena P. Sica A. (2002). Macrophage polarization: tumor-associated macrophages as a paradigm for polarized M2 mononuclear phagocytes. *Trends Immunol.* 7 549–555. 10.1016/s1471-4906(02)02302-5 12401408

[B34] Martinez F. O. Gordon S. Locati M. Mantovani A. (2006). Transcriptional profiling of the human monocyte-to-macrophage differentiation and polarization: new molecules and patterns of gene expression. *J. Immunol.* 177 7303–7311. 10.4049/jimmunol.177.10.7303 17082649

[B35] Mills C. D. Kincaid K. Alt J. M. Heilman M. J. Hill A. M. (2000). M-1/M-2 macrophages and the Th1/Th2 paradigm. *J. immunol.* 164 6166–6173. 10.4049/jimmunol.164.12.6166 10843666

[B36] Mizuno M. Iinuma M. Tanaka T. (1988). Flavonol glyeosides in the roots of *Epimedium diphyllum*. *Phytochemistry* 27 36–45. 10.1016/0031-9422(92)80024-9 1368039

[B37] Philippidis P. Mason J. C. Evans B. J. Nadra I. Taylor K. M. Haskard D. O. (2003). Hemoglobin scavenger receptor CD163 mediates interleukin-10 release and heme oxygenase-1 synthesis: antiinflammatory monocyte-macrophage responses in vitro, in resolving skin blisters in vivo, and after cardiopulmonary bypass surgery. *Circ. Res.* 94 119–126. 10.1161/01.RES.0000109414.78907.F9 14656926

[B38] Pollard J. W. (2004). Tumour-educated macrophages promote tumour progression and metastasis. *Nat. Rev. Cancer* 4 71–78. 10.1038/nrc1256 14708027

[B39] Porcheray F. Viaud S. Rimaniol A. C. Leone C. Samah B. Dereuddre-Bosquet N. (2005). Macrophage activation switching: an asset for the resolution of inflammation. *Clin. Exp. Immunol.* 142 481–489. 10.1111/j.1365-2249.2005.02934.x 16297160 PMC1809537

[B40] Quan X. Dujuan X. Zhaogang H. Jianjun L. Xinqun W. Xiu W. (2010). Preparation of icariside II from icariin by enzymatic hydrolysis method. *Fitoterapia* 81 437–442. 10.1016/j.fitote.2009.12.006 20026390

[B41] Shiraishi D. Fujiwara Y. Horlad H. Saito Y. Iriki T. Tsuboki J. (2018). CD163 is required for protumoral activation of macrophages in human and murine sarcoma. *Cancer Res.* 78 3255–3266. 10.1158/0008-5472.CAN-17-2011 29610117

[B42] Sica A. Bronte V. (2007). Altered macrophage differentiation and immune dysfunction in tumor development. *J. Clin. Invest.* 117 1155–1166. 10.1172/JCI31422 17476345 PMC1857267

[B43] Stein M. Keshay S. Harris N. Gordon S. (1992). Interleukin 4 potently enhances murine macrophage mannose receptor activity: a marker of alternative immunologic macrophage activation. *J. Exp. Med.* 178 287–292. 10.1084/jem.176.1.287 1613462 PMC2119288

[B44] Takeya M. Komohara Y. (2016). Role of tumor-associated macrophages in human malignancies: friend or foe: TAMs in human malignancies. *Pathol. Int.* 66 491–505. 10.1111/pin.12440 27444136

[B45] Thoennissen N. H. Iwanski G. B. Doan N. B. Okamoto R. (2009). Cucurbitacin B induces apoptosis by inhibition of the JAK/STAT pathway and potentiates antiproliferative effects of gemcitabine on pancreatic cancer cells. *Cancer Res.* 69 5876–5884. 10.1158/0008-5472.CAN-09-0536 19605406

[B46] Tsuboki J. Fujiwara Y. Horlad H. Shiraishi D. Nohara T. Tayama S. (2016). Onionin A inhibits ovarian cancer progression by suppressing cancer cell proliferation, and the protumour function of macrophages. *Sci. Rep.* 6:29588. 10.1038/srep29588 27404320 PMC4941721

[B47] Tu B. Du L. Fan Q. M. Tang Z. (2012). STAT3 activation by IL-6 from mesenchymal stem cells promotes the proliferation and metastasis of osteosarcoma. *Cancer Lett.* 325 80–88. 10.1016/j.canlet.2012.06.006 22743617

[B48] Wen-Kui L. Pei-Gen X. Jing-Qi P. (1998). Complete assainment of 1H and 13C NMR spectra of icarisoside A and epimedoside C. *Magn. Reson. Chem.* 36 303–304. 10.1002/mrc.2681 20821411

[B49] Yokogami K. Wakisaka S. Avruch J. Reeves S. A. (2000). Serine phosphorylation and maximal activation of STAT3 during CNTF signaling is mediated by the rapamycin target mTOR. *Curr. Biol.* 10 47–50. 10.1016/s0960-9822(99)00268-7 10660304

[B50] Yu H. Kortylewski M. Pardoll D. (2007). Crosstalk between cancer and immune cells: role of STAT3 in the tumour microenvironment. *Nat. Rev. Immunol.* 7 41–51. 10.1038/nri1995 17186030

[B51] Zhou J. Wu J. Chen X. Fortenbery N. (2011). Icariin and its derivative, ICT, exert anti-inflammatory, antitumor effects, and modulate myeloid derived suppressive cells (MDSCs) functions. *Int. Immunopharmacol.* 11 890–898. 10.1016/j.intimp.2011.01.007 21244860 PMC3109231

