# Supplementary material for: Flavonoid Compounds Contained in Epimedii Herba Inhibit Tumor Progression by Suppressing STAT3 Activation in the Tumor Microenvironment
Source: Front Pharmacol. 2020 Mar 18;11:262. doi: 10.3389/fphar.2020.00262 (PMC7093601; doi:10.3389/fphar.2020.00262)
Supplement: Supplementary file 1 [file Image_1.pdf]

Supplementary Material

1.1 Supplementary Figures

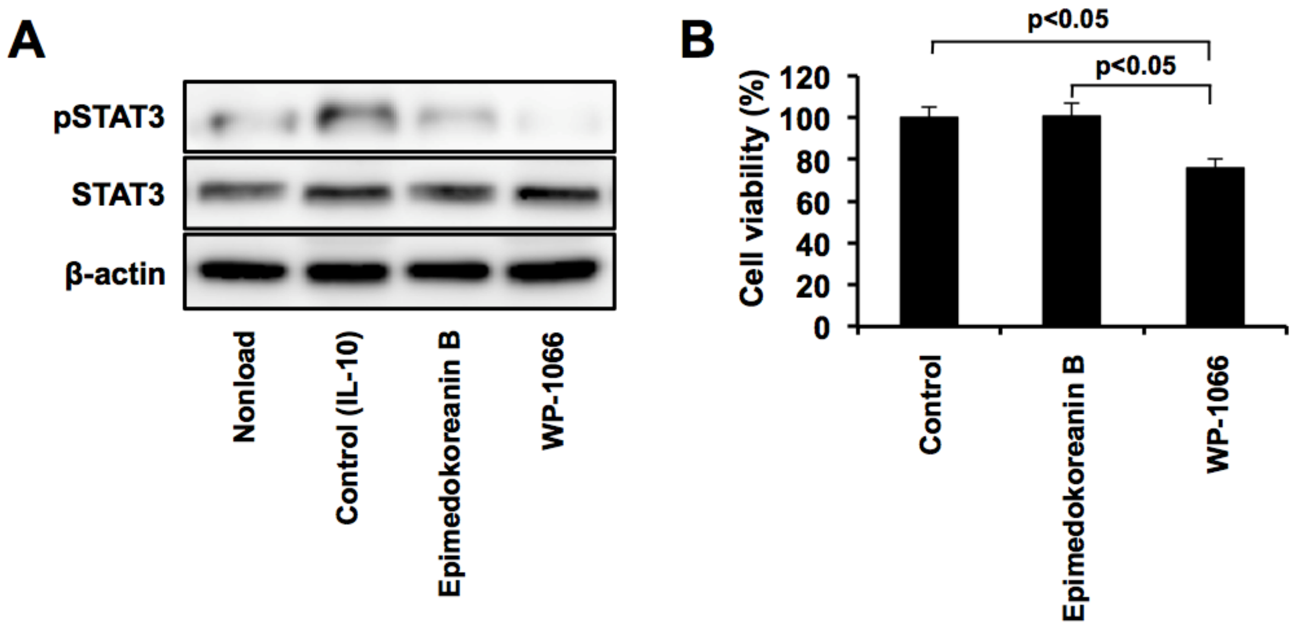

Supplementary Figure. Effects of STAT3 inhibitor on STAT3 activation and cell viability in human macrophages. Human monocyte-derived macrophages were incubated with WP1066 (5 µM), a STAT3 inhibitor, and epimedokoreanin B (5 µM) in the presence of IL-10 (20 nM) for 24 h, followed by determination of phosphorylated STAT3, STAT3, and β-actin expressions by western blot analysis (A). Human monocyte-derived macrophages were incubated with WP1066 (5 µM), a STAT3 inhibitor, and epimedokoreanin B (5 µM) for 24 h, followed by determination of cell viability using the WST-8 assay (B).
